# Uterine Tumor Resembling Ovarian Sex-Cord Tumor (UTROSCT): A Rare Polyphenotypic Neoplasm

**DOI:** 10.3390/diagnostics14121271

**Published:** 2024-06-17

**Authors:** Giovanna Giordano, Debora Guareschi, Elena Thai

**Affiliations:** Department of Medicine and Surgery, Pathology Unit, University of Parma, Viale A. Gramsci, 14, 43126 Parma, Italy; debora.guareschi@unipr.it (D.G.); elenathai@yaooh.com (E.T.)

**Keywords:** uterine tumor resembling ovarian sex-cord tumor, uterine mesenchymal neoplasm, polyphenotypic neoplasm, prognosis

## Abstract

Uterine tumor resembling ovarian sex-cord tumor (UTROSCT) is a rare form of uterine mesenchymal neoplasm. Although UTROSCT generally exhibits benign behavior with a favorable prognosis, this neoplasm is nevertheless classified as being of uncertain malignant potential, given its low rate of recurrence and the fact that it rarely produces metastases (e.g., in the lymph nodes, epiploic appendix, omentum, small bowel, subcutaneous tissue, lungs). Its histogenesis is also uncertain. Typically, UTROSCT occurs in peri-menopausal or menopausal women, but it can sometimes be observed in young women. Usually, this neoplasm can be found in the uterine corpus as a nodular intramural lesion, while it is less frequently submucosal, subserosal, or polypoid/intracavitary. UTROSCT can cause abnormal bleeding, pelvic pain, enlarged uterus, and mass sensation, but sometimes it is found purely by chance. This neoplasm can be considered polyphenotypic on morphological, immunohistochemical, and genetic analyses. Generally, upon microscopic examination, UTROSCT shows a predominant pattern of the cords, nests, and trabeculae typical of sex-cord tumors of the ovary, while immunohistochemically it is characterized by a coexpression of epithelial, smooth muscle, and sex-cord markers. The aim of this review is to report clinical and pathological data and genetic alterations to establish their impact on the prognosis and management of patients affected by this rare entity.

## 1. Introduction

Uterine tumor resembling ovarian sex-cord tumor (UTROSCT) is a rare mesenchymal neoplasm of the uterus, accounting for less than 0.5% of all uterine malignancies and 10–15% of mesenchymal uterine malignancies [1].

Morphologically, this neoplasm resembles ovarian sex-cord tumors, without any component recognizable as an endometrial stroma. In 1945, Morehead and Bowman first described a case of UTROSCT as a uterine neoplasm resembling a granulosa cell tumor of the ovary [2]. Later, in 1976, Clement and Scully described this entity as a uterine neoplasm characterized by the presence of a component with sex-cord differentiation, and they subdivided it into two groups based on morphological and prognostic features [3]. Group I corresponded to endometrial stromal tumors with foci of sex-cord differentiation (ETSCLEs) < 50%, associated with recurrences and metastases, while Group II was composed predominantly or exclusively of sex-cord-like elements and was named uterine tumors resembling ovarian sex-cord tumors (UTROSCTs). According to the current World Health Organization (WHO) classification, UTROSCT is included under “Miscellaneous mesenchymal tumors,” and it is still considered to be a uterine neoplasm with a component that resembles those seen in ovarian sex-cord tumors, but without any component recognizable as endometrial stroma [4].

The histogenesis of these rare neoplasms is still unknown. However, many theories about the histogenesis of UTROSCTs have been suggested. According to some authors, this neoplasm could arise from ovarian sex-cord cells that have been displaced during embryogenesis. Conversely, others think that UTROSCTs could arise from an uncommitted mesenchymal stem cell, from overgrowth of sex-cord elements within an endometrial stromal neoplasm, adenosarcoma, or within foci of adenomyosis and endometriosis [3,5,6,7,8,9,10]. However, molecular studies have demonstrated that UTROSCTs have no molecular alterations found in endometrial stromal tumors, such as JAZF1–JJAZ1 or PHF1 fusion [7]. In addition, although UTROSCTs have a similar morphology to ovarian adult granulosa cell tumors and sex-cord stromal tumors, they do not present the same mutations that can be observed in these neoplasms, such as DICER1 and FOXL2 mutations [11,12]. The association with the use of tamoxifen for the treatment of breast carcinoma and UTROSCTs in some cases reported in the literature could suggest a correlation with this drug [13,14,15,16,17,18]. Because of its rarity and peculiar morphological findings, UTROSCTs can pose many problems for pathological diagnosis. The aim of this review is to report clinical and pathological data and genetic alterations to establish their impact on the prognosis and management of patients affected by this rare entity.

## 2. Clinical Data

As regards age, this neoplasm often occurs in peri-menopausal or menopausal women [19]. However, in a more recent and large series reported by Boyraz et al., the patients’ age ranged from 21 to 84 years (mean: 52.4; median: 53) [20]. Moreover, in the literature, many cases have been reported in which the patients were aged < 40 years [16,21,22,23,24,25,26,27,28,29,30,31,32] or even very young women [33,34]. The most common symptom found is abnormal vaginal bleeding (67.1%) [35]. Other symptoms observed include pelvic pain (5.1%) and palpable pelvic masses (2.5%) [35]. In rare instances, extra-uterine symptoms have been observed due to hormonal alterations. Thus, in a case reported by Dimitriadis, the presence of UTROSCT was associated with hyperprolactinemia and galactorrhea, which resolved after tumor resection [36]. Moreover, in the case described by Suzuki et al., a patient with UTROSCT had elevated serum calcium and parathormone (PTH-rP) levels, and in this case the tumor’s production of PTH-rP was demonstrated by normalization of serum PTH-rP after a tumorectomy, as well as by the presence of immunoreactivity for PTHrP in the tumor cells [37]. In other cases, UTROSCT can be asymptomatic and may be found by chance [15,20,35].

Most tumors exhibit benign behavior. In fact, we have found small case series in which the patients were free of recurrences and metastases [15,20,32,38]. As a result, there are many examples of UTROSCT being treated with conservative surgery [16,22,23,24,25,26,27,28,29,30,31,32,39,40,41], and in only three of these cases [30,31,41] was recurrence observed after conservative surgery, which could well have been due to previous incomplete surgery. Thus, as suggested by Watrowski et al. and Carbone et al., in young women, fertility-sparing surgery should be offered to patients who wish to preserve their fertility [28,32]. However, it is important to advise such patients that the neoplasm may recur [25].

In addition, close follow-up should be implemented after conservative surgery, while radical surgery should be considered after a pregnancy [28,32].

In this review, we observed that many cases of UTROSCT that recurred or developed metastases were observed in single case reports [27,31,35,42,43,44,45,46,47,48,49,50,51,52,53] (Table 1) or sometimes in very small case series [19,30,40,54,55,56] (Table 2).

The most frequent sites of recurrences or metastases in these cases were the lymph nodes, peritoneum, omentum, vaginal vault, lungs, [44,45,47,48,50,51,52,53]. Interestingly, in these single case reports and small case series, there are also examples where the recurrences and the metastases were observed many years later when diagnosing lymph nodes and liver [56] in the pelvis and omentum [19] (Table 1 and Table 2).

More recently, from 2017 onwards, we found five only studies with larger series, and for these it was possible to establish a recurrence or metastasis rate [8,20,41,57,58] (Table 3).

In their study, Moore and McCluggage found that 8/34 tumors behaved aggressively, as defined by either lymph node metastatic disease at diagnosis (*n* = 1) or recurrence (*n* = 7), indicating a rate of 23.5%, with a follow-up ranging from 6 to 135 months (mean: 39) [7] (Table 3). Moreover, in this study, three patients (23.5%) developed metastases in the liver, vertebra, and clavicle, respectively, after 12–23 months and died from their disease [8] (Table 3).

In the series of Goebel et al., follow-up information was available for 11 out of 26 patients (42.3%), with a mean follow-up interval of 94.4 (range: 1–319) months, and in only one case did the neoplasm recur in the pelvis (66 months after the initial diagnosis) [57] (Table 3). In a study by Boyraz et al., only a minority of the cases showed a malignant outcome (5/58, 8.6%). In this work, 5 out of 58 patients followed up (22 to 192 months; mean: 73.2) had recurrences/metastases from 30 to 144 months, and 2 died from the disease. Out of three cases with metastases, in one case the metastasis was pulmonary, and this was observed at the time of diagnosis. In the remaining two cases, the metastases developed 60 and 48 months after diagnosis, respectively, involving the peritoneum, brain, and femur. In these cases, death occurred 96 and 50 months after diagnosis, respectively [20].

In a study by Bi et al., there were 7/22 (31.81%) cases with recurrences or metastases, and they involved the pelvic lymph nodes, pelvis, and omentum, and in one case the abdominal aorta. Death was observed in two cases, 177 and 44 months after diagnosis, and in one of these cases the patient already had pelvic lymph node involvement at diagnosis [41] (Table 3).

Xiong et al., in their analysis of 19 cases, observed that six patients (31.6%, 6/19) had tumor recurrences, with a median follow-up of 40.9 months (range: 1.2–195.3 months) [58]. One case was excluded due to molecular translocation suggesting an endometrial stromal neoplasm (JAZF1–SUZ12) [7,59] (Table 3). The sites of these recurrences and metastases were the peritoneum, pelvis, colon, and lungs. Only one patient died, 26 months after diagnosis. It is extremely interesting to note that the recurrences were observed in two cases many months after diagnosis (144 and 195 months, respectively) [58] (Table 3).

## 3. Pathological Features

For the diagnosis of UTROSCT, there are no specific imaging findings, so it is only possible to establish that a uterine lesion corresponds to a UTROSCT through pathological examination along with accurate morphological and immunohistochemical analysis.

### 3.1. Macroscopic Findings

Upon macroscopic examination, UTROSCT is often located in the uterine corpus and can be an intracavitary polypoid lesion mimicking an endometrial polyp that can be removed by hysteroscopy [23,59] or it can appear as a pale-yellow submucosal mass located within the thick muscle [15,23,27,30,31,34,45,46,54,60]. UTROSCT can also be observed as a yellowish-white intramyometrial mass located in the uterine corpus [34,36,41,45,46,48,50,51,52,53,54].

Sometimes, the lesion can have hemorrhagic cystic necrotic areas [42], or it may be a yellow cystic/solid mass [41,44], and in rare occurrences it can even appear as a subserosal peduncle solid lesion attached to the uterine fundus [55,61,62], mimicking a subserosal leiomyoma. In addition, when the neoplasm shows prominent myxoid features with a prominent gelatinous appearance on gross examination, a diagnosis of myxoid leiomyoma or leiomyosarcoma [63] can be suggested. As reported by Liu et al., the lesion can also be located at the isthmus and can protrude through the cervical os [40].

More rarely, a UTROSCT has been observed in the cervix. In fact, in the literature, we found only four cases [21,33,37,64] that presented as cervical masses, mimicking a primary cervical carcinoma on instrumental tests such as computed tomography (CT) or pelvic magnetic resonance imaging (MRI), as well as on macroscopic, histological, and cytological examination. Thus, as emphasized by Dubruc et al., it is important to keep in mind that UTROSCTs can also be encountered in current cervical screening programs, in which case they can be responsible for diagnostic pitfalls [64].

### 3.2. Microscopic Findings

**Figure 1:**
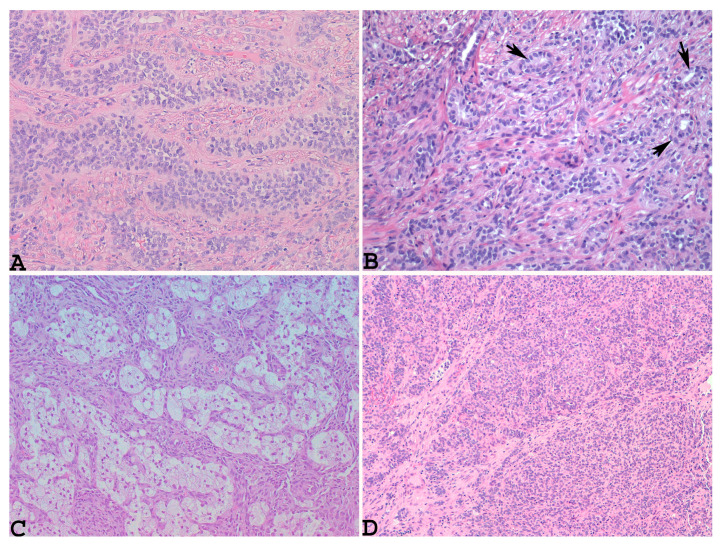
Microscopically, UTROSCT resembles ovarian sex-cord stromal tumors, with sheets, cords, nests, and trabeculae ((**A**): hematoxylin–eosin ×200) or tubules ((**B**): hematoxylin–eosin, blackarrows: tubules, ×200), along with scattered foam cells, consisting of single cells and small or larger aggregates of cells with round, central nuclei and abundant clear-to-foamy cytoplasm resembling foam cell macrophages or Sertoli cells ((**C**): hematoxylin–eosin, ×100) and glomeruloid structures ((**D**): hematoxylin–eosin, ×100) (personal cases observed during diagnostic activity of G.G. and E.T. at the Department of Medicine and Surgery, Parma University, Italy).

Given the rarity of this type of tumor, the diagnosis of UTROSCT is usually made postoperatively through histopathological and immunohistochemical analyses. Typically, such neoplasms resemble ovarian sex-cord stromal tumors, with sheets, cords, nests, and trabeculae (Figure 1A) or tubules (Figure 1B) [8]. Neoplastic cells are epithelioid with scant eosinophilic cytoplasm, and their nuclei are bland with minimal atypia (Figure 1A,B).

Necrosis and mitoses are rare or absent [65]. Sometimes, the neoplasm may show scattered foam cells, consisting of single cells and small or larger aggregates of cells with round, central nuclei and abundant clear-to-foamy cytoplasm resembling foam cell macrophages or Sertoli cells [34,66] (Figure 1C). Glomeruloid structures (Figure 1D) and small nests or micropapillary-like structures [15,16,59,61,67] along with micro-follicles resembling Call–Exner bodies [20,59] can also be obserOnly one patient died, month Vr yeaus after diagnosis. It is extremely interesting to note that the recurrences. Another peculiar growth pattern is that of a retiform type, with labyrinthine cavities and channels resembling the rete ovarii [15,61]. Sometimes, this pattern can be prominent, mimicking an adenocarcinoma or myometrial metastasis from a previous breast cancer on small endometrial biopsy [15]. Goebel et al. also observed microcystic and signet ring-like cell changes and retiform patterns [57]. Rhabdoid cells with abundant dense eosinophilic cytoplasm and eccentric nuclei were found to be diffuse in the examples reported by Boyaz et al. and Bennett et al. [19,20]. Rhabdoid cells with a single-file growth pattern have been reported by other authors [51,61]

However, many patterns can be present within the same neoplastic mass, causing considerable morphological heterogeneity [15,57,59,68,69].

When the neoplasm shows a predominant tubular pattern or gland-like differentiation with few associated stromal characteristics, this can frequently pose diagnostic problems, mimicking sertoliform endometrial adenocarcinoma [70,71,72] or extragenital metastatic carcinoma [73,74]. In addition, when there is both a tubular pattern and lipid-rich cells, UTROSCT can imitate a Sertoli tumor [42].

**Figure 2:**
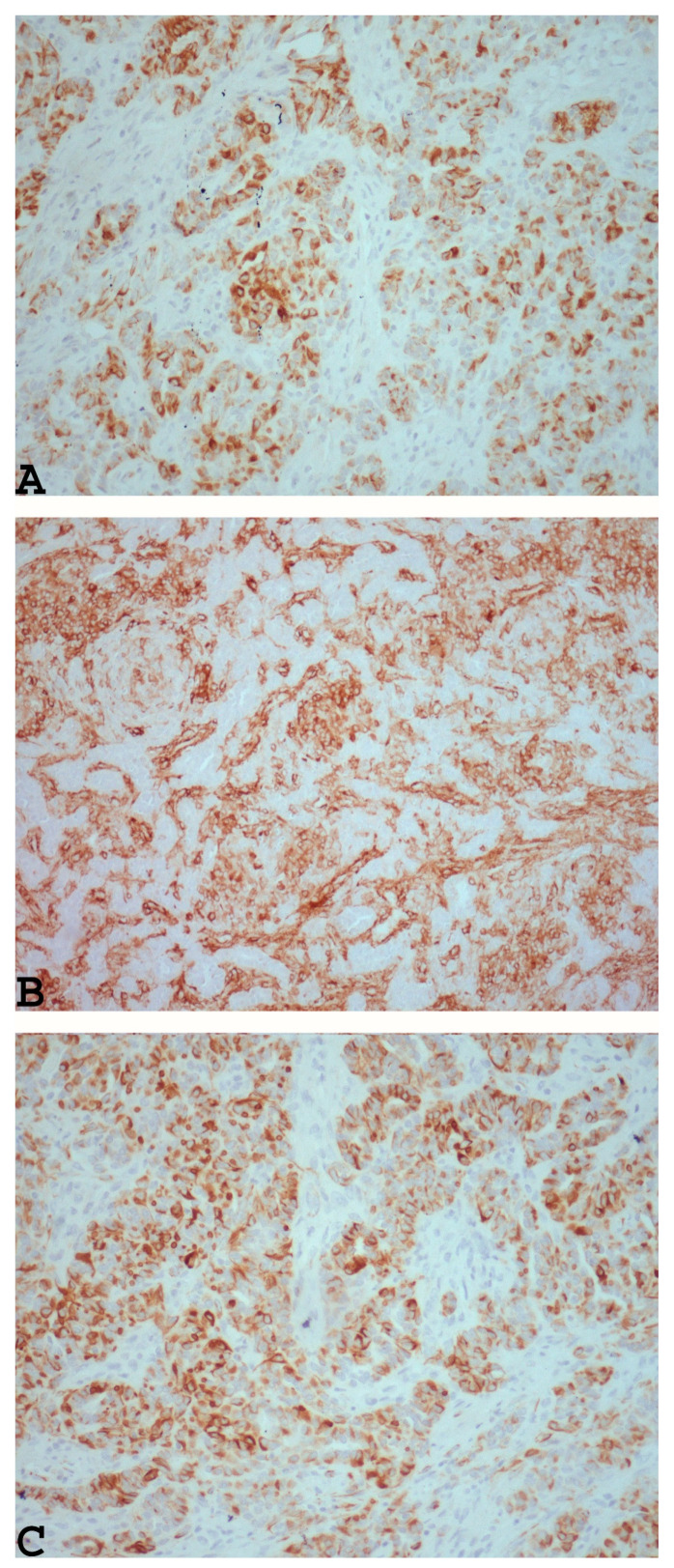
Example of UTROSCT exhibiting polyphenotypic immunoreactivity with co-expression of cytokeratin ((**A**): ×200) and smooth muscle markers, including muscle actin ((**B**): ×200) and desmin ((**C**): ×200) (personal cases observed during diagnostic activity of G.G. and E.T. at the Department of Medicine and Surgery, Parma University, Italy).

Upon immunohistochemical analysis, UTROSCTs characteristically exhibit polyphenotypic immunophenotypes, with co-expression of cytokeratin (Figure 2A) and smooth muscle markers, including muscle actin (Figure 2B), desmin (Figure 2C), and histone deacetylase 8 hormone receptors [61].

**Figure 3:**
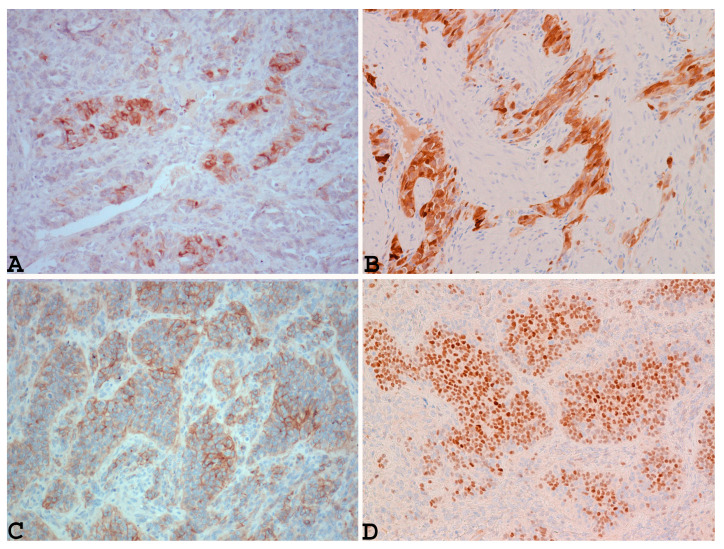
Example of UTROSCT with positivity for markers that are commonly positive in ovarian sex-cord stromal neoplasms, such as inhibin ((**A**): ×200), calretinin ((**B**): ×200), CD99 ((**C**): ×200), and nuclear staining with steroidogenic factor 1 (SF-1) ((**D**): ×200) (cases observed during diagnostic activity of G.G. and E.T. at the Department of Medicine and Surgery, Parma University, Italy).

In addition, this neoplasm shows positivity for markers that are commonly positive in ovarian sex-cord stromal neoplasms, such as inhibin (Figure 3A), calretinin (Figure 3B), CD99, Wilms tumor protein 1 (WT1), and MART-1 [16,59,61,65], as well as other markers such as CD10, S100, and CD117 [60,66].

In addition, as reported by Croce et al., UTROSCT can characteristically show nuclear staining with steroidogenic factor 1 (SF-1) (Figure 3D) [51], similarly to ovarian sex-cord tumors (Figure 3D).

Moreover, other authors [61,75] inadvertently found a strong, diffuse BCL-2 positivity in all reported cases, which could be related to peculiar genetic alterations consisting of translocations on chromosomes t(4; 18) (q21.1; q21.3) and t(x; 6) (p22.3; q23.1) [75]. Consequently, they suggested that this marker could also have potential value in the diagnosis of UTROSCT. These peculiar immunohistochemical aspects of this neoplasm are of considerable importance in differentiating UTROSCT from other uterine neoplasms that could present morphological similarities. The most frequent uterine neoplasms that could mimic UTROSCT and should be considered are histopathological variants of leiomyomas, such as leiomyoma with tubules, epithelioid leiomyoma, or vascular plexiform leiomyoma. Uterine leiomyoma with tubules is a biphasic neoplasm composed of epithelial and mesenchymal elements, with intersecting fascicles of smooth muscle, along with tubular and gland-like structures lined by plump cells with indistinct cytoplasm [76]. Thus, histologically, this type of lesion can simulate uterine tumors resembling ovarian sex-cord tumors (UTROSCTs), but the immunophenotype is not consistent with true sex-cord differentiation in its negativity for inhibin, CD99, CD10, and Melan A [76]. Epithelioid leiomyoma is a subtype of leiomyoma that macroscopically appears as a well-circumscribed, intramural mass with a soft consistency and yellow-to-tan cut surfaces. Microscopically, it is characterized by the presence of more than 50% round–polygonal cells, and immunohistochemically it shows immunoreactivity to epithelial and smooth muscle markers, but negativity for typical sex-cord markers of UTROSCT [77].

Histologically, vascular plexiform leiomyoma is a well-circumscribed, intramural nodule with anastomosing cords and trabeculae of two to three cell layers with eosinophilic cytoplasm, indistinct cell borders, and plump, slightly hyperchromatic nuclei. In addition, its cord lumens contain red blood cells. On immunohistochemical analysis, the neoplastic cells in this subtype of leiomyoma are positive for smooth muscle actin (SMA), caldesmon, and CD99, but they are negative for inhibin α [78].

Another uterine neoplasm that could mimic UTROSCT is a low-grade endometrial stromal sarcoma (LGESS) in a biopsy or curettage specimen. This malignancy represents the second-most common uterine sarcoma [79] affecting a wide age range, but with a predilection for pre-menopausal and peri-menopausal women [80]. Moreover, LGESS, like UTROSCT, may be related to tamoxifen treatment [81]. Histologically, LGESS is composed of permeative tongue-like islands of tumor cells consisting of monotonous oval to spindle cells with minimal cytological atypia and a whorl pattern of growth around blood vessels. Immunohistochemical analysis is particularly useful here, since LGESS shows positivity for CD10 and negativity for sex-cord markers [82]. Moreover, these endometrial sarcomas, along with ESTSCLE, show genetic alterations such as JAZF1–JJAZ1 or PHF1 fusion that are absent in UTROSCT [7].

Plexiform tumorlets are rare tumors affecting patients with an average age of 48 to 60 years. A plexiform tumorlet is a rare type of lesion that is usually found in the myometrium and is considered to be a variant of epithelioid leiomyoma. Multiple plexiform tumorlets may have an infiltrative pattern and mimic endometrial stromal sarcoma.

Uterine sertoliform endometrioid adenocarcinoma is a rare subtype of endometrial carcinoma that can mimic UTROSCT on morphological [70,71] and immunohistochemical analyses due to the presence of tubules and glandular structures, along with its positivity for sex-cord markers such as inhibin, CD99, calretinin, WT-1, and Melan A [72]. In such an occurrence, for a correct differential diagnosis between UTROSCT and uterine sertoliform endometrioid adenocarcinoma, it is important to keep in mind that the remaining endometrium in this subtype of endometrial adenocarcinoma is affected by atypical complex hyperplasia [72], and a typical endometrioid component can be observed within the neoplasms [70].

### 3.3. Electron Microscopy Findings

In addition, upon ultrastructural analysis, UTROSCT exhibits polyphenotypic features, with both epithelial structures (such as desmosome-like junctions, tonofilaments, lumina formation, and microvilli) and sex-cord-like features (including nuclear indentation, abundant intracellular filaments, sparse-to-moderate rough endoplasmic reticula, and abundant intracytoplasmic lipids) [83].

## 4. Impact of Pathological Features on Recurrences or Metastases in UTROSCT

Given that there are many single case reports of UTROSCT in the literature (Table 1) [27,31,35,36,42,43,44,45,46,47,48,49,50,51,52,53], along with small case series [19,20,40,54,55,56] (Table 2) and a few lager case series [8,20,41,57,58] (Table 3), in our opinion, it is extremely difficult to establish which morphological aspects could accurately predict its aggressive behavior and poor prognosis.

Moreover, in some cases with recurrences or metastases, morphological findings that could be signs of malignancy, such as size [8,19,30,49,52,54], LVI [19,27,30,31,36,44,49,50,51,52,53,54,55,56], necrosis [19,27,30,36,40,43,48,49,50,51,54,55,57], number of mitoses [19,27,30,40,41,46,49,55,58], or nuclear atypia [27,30,31,35,36,40,44,46,48,49,50,52,53,54,55,56,58] not always reported (Table 1, Table 2 and Table 3). In addition, in the small series reported by some authors, it seems that there were cases that recurred or caused metastases, even though they presented benign morphological features, such as well-defined margins [40,41,42,51] (Table 2), absence of necrosis [8,19,31,35,41,46,52,54,56,58], a low or insignificant number of mitoses [8,19,20,31,35,41,42,43,44,47,50,51,52,53,54,56,57,58], absence of nuclear atypia [8,31,42,43,45,46,47,51,52,53,54,56,58], and absence of LVI [8,19,20,35,40,42,43,45,56,58] (Table 1, Table 2 and Table 3).

Regarding their microscopic appearance, the neoplasms did not show any particular architectural pattern. In fact, the majority of the cases reported in the literature that recurred or developed metastases revealed the presence of cords, trabeculae, nests, or tubules [20,30,31,36,40,50,52,53,56,57,58], except for one case reported by Croce et al., which also presented focal rhabdoid cells [51], and a case observed by Bennett that was characterized by the presence of an extensive rhabdoid component [19].

Although there are no clear criteria to establish a firm prognosis or malignancy level for UTROSCT, more recently, Boyraz et al., in their large study with 75 cases, considering all morphological features, and with follow-up available for 58 women, affirmed that it is important to simultaneously evaluate many features for every neoplasm. In fact, these authors observed that malignant tumors that developed recurrences/metastases, compared with benign neoplasms, showed more than three of the following five features: size > 5 cm, moderate nuclear atypia, >3 mitoses per 10 high-power fields (HPFs), infiltrative borders, and necrosis, and probably also an extensive rhabdoid component [20]. In addition, Boyraz et al. emphasized that sometimes it is impossible to define the prognosis of UTROSCT, since occasionally the entire neoplasm is not examined upon microscopic examination, but only some fragments from curettage specimens. Thus, it is perfectly possible that some features of malignancy, such as infiltration of myometrial tissue, could be missed [20].

## 5. Molecular Alterations of UTROSCT and Its Impact on Prognosis

In this review, we evaluated the presence of molecular alterations in order to establish whether they could have an impact on the prognosis of UTROSCT. We found a few recent studies that reported using fluorescence in situ hybridization (FISH) and RNA sequencing validated by RT-PCR [19,41,51,52,53,57,58] Table 1, Table 2 and Table 3), [69,75,84].

Wang et al., in a single case report using fluorescence in situ hybridization (FISH), observed two balance translocations in cultured cells: t(4; 18) (q21.1; q21.3) and t(X; 6) (p22.3;q23.1) [75]. The translocations t(4; 18) (q21.1; q21.3) are related to the bcl2 gene and the development of particular tumors, such as more aggressive squamous-cell carcinomas and some forms of acute leukemia or follicular lymphomas. The translocation t(X; 6) (p22.3; q23.1) instead involves the antigen regulator gene (H-Y R), which is located at p22.3 and is responsible for gonadal organogenesis. However, the molecular results in this study did not provide information on prognostic significance, since the patient was well after a short follow-up of 12 months, with no signs of disease [75]. On the other hand, Croce et al., in a 70-year-old patient affected by UTROSCT with a ruptured uterine serosa and a focal rhabdoid component that recurred with widespread pelvic nodules 17 months after surgery and then developed lung metastases 1 year later despite treatment with aromatase inhibitors, demonstrated a novel translocation, t(2;3) (p25; p22), involving GREB1 (intron 8) and CTNNB1 (exon 3), using RNA sequencing validated by RT-PCR. This peculiar molecular alteration, observed in both primary and recurrent neoplastic tissue, was responsible for nuclear overexpression of hypophosphorylated and truncated beta catenin, which, thanks to the involvement of GREB1, was produced in response to estrogens and caused the activation of the Wtn/beta catenin signaling pathway, with a major oncogenic effect [51].

In 2019, Dickson et al., using RNA sequencing confirmed by FISH in four cases of UTROSCT, first observed that this entity presents peculiar genetic alterations [69]. 

These genetic alterations, when identified, corresponded to *NCOA2–3* gene fusions in four cases of UTROSCT, including *ESR1–NCOA3 (n = 2)*, *ESR1–NCOA2 (n = 1)*, and *GREB1–NCOA2 (n = 1)* [69].

Characteristically, these neoplasms did not reveal conspicuous mitotic activity. On the contrary, one tumor was circumscribed, while the remaining three cases showed myometrial infiltration as a low-grade endometrial stromal sarcoma. The authors, given the genetic alterations found, suggested that these could be used for an accurate diagnosis of UTROSCT [69].

In our opinion, although the authors suggested that these genetic alterations may be related to malignant mesenchymal neoplasms such as mesenchymal chondrosarcoma [85] congenital spindle-cell rhabdomyosarcoma [86,87], alveolar rhabdomyosarcoma [88], Ewing sarcoma [89], or human leukemia [90], as well as some uterine sarcomas with variable sex-cord differentiation [91,92], this study is not useful to establish the prognostic significance of these genetic alterations, because no information was reported about the follow-up of the patients [69].

More recently, it has been demonstrated in some studies that UTROSCTs with growth-regulating estrogen receptor-binding 1 (GREB1) rearrangement may display more aggressive biological behavior, with high risks of recurrence or metastases [52,57]. Likewise, it seems that GREB1-rearranged tumors tend to be larger and more mitotically active [52].

Additionally, neoplasms with estrogen receptor 1 *(ESR1)–NCOA2* fusions are more likely related to recurrences and the presence of infiltrative margins, and sometimes to the presence of an extensive rhabdoid component, as demonstrated by Bennett et al. [19]. Furthermore, tumors with *GREB1–NCOA2* fusion could be more frequently related to recurrences than those with ESR1 Rearrangement [41,51].

These data suggest that these gene fusions probably cause aberrant activation of estrogen signaling pathways, with a major oncogenic effect due to the increase in the proliferation and activation of neoplastic cells. In fact, gene fusions involving three nuclear receptor coactivators, such as *NCOA1*, *NCOA2*, and *NCOA3*, have been demonstrated in many cases of UTROSCT [53,57]. The *NCOA* genes belong to a p160 family of steroid receptor coactivators, which interact with ligand-dependent hormone nuclear receptors, including estrogen receptor alpha (ERα), to mediate transcriptional programs, enabling them to promote a wide range of signaling pathways, including cellular proliferation, metabolism, growth, and survival [90].

In our opinion, a recent study conducted by Xiong et al. is very interesting. The authors considered programmed cell death ligand 1 (PDL1), which is a transmembrane protein, a co-inhibitory factor of the immune response, and plays an important role in various malignancies, attenuating the host immune response to tumor cells. First, they correlated the expression of this marker with mitotic activity and *NCOA2* gene alterations in a small series of UTROSCTs [58]. Thus, they discovered that UTROSCTs with significant mitotic activity, gene alteration of *NCOA2*, and a high expression of stromal PDL1 represent the subset of this neoplasm with aggressive behavior and shorter disease-free survival [58]. Consequently, Xiong et al. suggested that UTROSCTs with aggressive behavior have a peculiar tumor microenvironment and could be treated with immunotherapy in line with other neoplasms [58].

## 6. Conclusions

In conclusion, through our review of the literature, we have determined that UTROSCT is a rare neoplasm. In our opinion, its pathological diagnosis can be difficult for many reasons. First, the neoplasm is rare, and often the diagnosis must be made by an expert in the field. In addition, due to the many histological features that the lesion can show, many differential diagnoses should be considered when the lesion presents as a uterine mass. Moreover, similar to many uterine intra-cavitary and intramural lesions, the most common symptom of this neoplasm is abnormal vaginal bleeding. Thus, this symptom is not useful for diagnosis. To the best of our knowledge, for pre- or perioperative diagnosis of UTROSCT, there are no studies that have evaluated serum inhibin levels (a marker of sex-cord differentiation). Moreover, imaging modalities such as MRI cannot be useful to identify this neoplasm because they provide findings such as cystic degeneration, intratumoral hemorrhage, and necrosis, which are common to leiomyoma [93,94,95] or adenomyosis [32,96]. However, for a pathological diagnosis, many studies over the past few years have contributed to improving pathological diagnosis via immunohistochemical analysis with specific markers, revealing that UTROSCT is a polyphenotypic neoplasm with variable positivity for epithelial, smooth muscle, neuroendocrine, and sex-cord markers and hormone receptors, along with molecular heterogeneity [19,41,51,52,58,84].

Although it is extremely difficult to establish which morphological aspects of this rare neoplasm could predict its aggressive behavior, some authors have recently suggested evaluating multiple factors [20]. More recently, many molecular biology studies have revealed that UTROSCTs with *GREB1–NCOA1–3* fusions [8,52] and PDL1 molecule expression appear to be predisposed to more aggressive behavior and recurrences, with *GREB1–NCOA2* being the most common gene fusion in recurrent tumors [58].

Additionally, Yin et al. recently observed a case of UTROSCT with aggressive histological features harboring a *GREB1–NCOA2* fusion, such as increased mitotic figures (up to 3 per 10 high-power fields), geographic necrosis, and LVI [97].

Thus, it is especially important in the case of this peculiar neoplasm to perform molecular investigations in order to define the most aggressive forms and to select patients with a higher risk of recurrences or metastases. In our opinion, it is also very important for every patient to receive a long and accurate follow-up because, as revealed in this review, many recurrences can occur many months after diagnosis. Further studies with more numerous cases should be carried out to correlate morphological findings, molecular data, and clinical data in order to identify subtypes with a worse prognosis.

## Figures and Tables

**Table 1:** Single case reports of UTROSC with recurrences or metastases: clinical, pathological, and molecular features.

Authors and Year	Age (Years)	Gross Appearance	Tumor Size (cm)	Common Microscopic Appearance	Microscopic Findings of Malignancy	Molecular Findings	Site and Time of Recurrence/ Metastasis
Kantelip et al., 1985 [42]	86	Multiple myometrial cyst with hemorrhagic and necrotic fluid	2–10	Yes	Necrosis	NR	Ovary and epiploon at diagnosis
Biermann et al., 2007 [43]	68	Grayish-yellow nodule	4.5	Yes	Margins focally infiltrative	NR	Mesentery and small bowel (48 months)
O’Meara et al., 2009 [44]	35	Solid cystic yellow mass	9.9	Yes	Serosal infiltration	NR	Bladder, ovaries, abdominal intestinal wall, lymph nodes (36 months)
Mačák et al., 2014 [45]	53	Polypoid myometrial mass	1.5	Yes	Absent	NR	Lymph nodes at diagnosis
Jeong et al., 2015 [27]	32	Submucosal protruding mass	3.6	Yes	Infiltrative margins	NR	Local recurrence (17 months)
Gomes et al., 2015 [46]	53	Myometrial mass	12	Yes	Infiltrative margins, LVI, necrosis	NR	Parametrium, ovary at diagnosis
Endo et al., 2016 [47]	62	NR	NR	Yes	Myometrial infiltration	NR	Pelvic lymph nodes 23 years
Kuznicki et al., 2017 [48]	49	Intra-myometrial mass	6	Yes	LVI, high KI67	NR	Death 15 months, liver peritoneum pelvis 4 months
Kondo et al., 2018 [49]	69	NR	NR	NR	NR	NR	Lung (36 months)
Cömert et al., 2018 [35]	61	Endometrial–myometrial lesion	7	Yes	Infiltrative margins	NR	Pelvis abdominal (60 months) (75 months)
Marrucci et al., 2019 [50]	54	Grayish-yellow mass	9	Yes	NR	NR	Vaginal vault (59 months)
Croce et al., 2019 [51]	70	Well-circumscribed, yellow mass	10	Focal rhabdoid cells	Serosal infiltration	*GREB1–CTNNB1* fusion	Pelvis (17 months) Lung and peritoneum (29 months)
Chang et al., 2020 [52]	57	Well-circumscribed, soft, yellow mass	10	Yes	Infiltrative margins	*GREB1–NCOA2* fusion	Pelvis (30 months)
Dimitriadis et al., 2020 [36]	46	Well-circumscribed uterine mass	11	Yes	6 mitosis/10 HPF	NR	Intraabdominal recurrence (24 months)
Devereaux et al., 2021 [53]	42	Myometrial mass	8	Yes	Necrosis	*GFT2A1–NCOA2* fusion (in recurrent tumor)	Serosal surface of uterus, ovaries, large bowel, anterior abdominal wall (6 months)
Dondi et al., 2021 [31]	24	Submucosal mass	3	NR	Absent	NR	Local recurrence (20 months)

Table Legend: HPF: high-power field; LVI: lymphovascular invasion; NR: not reported.

**Table 2:** Small series of UTROSC with recurrences or metastases: clinical, pathological, and molecular features.

Authors and Year (Malignant Cases/Total of Cases)	Age (Years)	Gross Appearance	Tumor Size (cm)	Common Microscopic Appearance	Microscopic Findings of Malignancy	Molecular Findings	Site and Time of Recurrence/ Metastasis
Umeda et al., 2014 (2/2 cases) [54]	38	Yellowish-white intramyometrial mass	4.5	Yes	Infiltrative margins/necrosis	NR	Lymph node metastasis at diagnosis
Liu et al., 2015 (1/4 cases) [40]	50	Isthmus mass protruding through cervical os	4.5	NR	Absent	NR	Local recurrence (10 months)
Schraag et al., 2017 (2/3 cases) [30]	24	Submucosal nodule	NR	NR	NR	NR	Local recurrence (9 months)
Viau et al., 2017 (1/2 cases) [55]	43	Subserosa pedunculated solid mass whitish-yellow with ruptured surface	13 and 5.5	Yes	NR	NR	Pelvis (40 months). Sigmoid serosa and peritoneal implants at diagnosis.
Kaur et al., 2020 (1/6 cases) [56]	47	Mass in the uterine body	9.3	Yes	Infiltrative margins	NR	Pelvis, retroperitoneal lymph nodes, lungs (7 months)
Bennett et al., 2020 (3/3 cases) [19]	37	Yellow white myometrial mass	NA	Extensive rhabdoid component	Infiltrative margins Suspect LVI	*ESR1-NCOA2* fusion	Pelvis (7 years)

Table Legend: LVI: lymphovascular invasion; NR: not reported.

**Table 3:** Larger series of UTROSC with recurrences or metastases: clinical, pathological, and molecular features.

Authors and Year (Malignant Cases/Total of Cases)	Age (Years)	Gross Appearance	Tumor Size (cm)	Common Microscopic Appearance	Microscopic Findings of Malignancy	Molecular Findings	Site and Time of Recurrence/ Metastasis
Moore and McCluggage, 2017 (8/34 cases) [8]	44	NR	12.5	NR	Absent	NR	Para-aortic lymph nodes, peritoneum (11 months)
	75	NR	NA	NR	Absent	NR	Para-aortic lymph nodes, retroperitoneal and sacral metastasis present at diagnosis
	62	NR	7	NR	Necrosis	NR	Peritoneum, lung (33 months)
	43	NR	1	NR	Absent	NR	Pelvis, peritoneum (25 months)
	47	NR	6	NR	Necrosis	NR	Vertebra, ovary (78 months)
	68	NR	8	NR	Absent	NR	Death (12 months) Peritoneum, liver (11 months)
	61	NR	12.5	NR	LVI, necrosis	NR	Death (23 months) Vertebra, clavicle (12 months)
	72	NR	7	NR	Absent	NR	Death (23 months), liver (23 months)
Goebel et al., 2020 (1/26 cases) [57]	74	NR	13	Yes	Infiltrative margins	*GREB1–NCOA2* fusion	Pelvis (66 months)
Boyraz et al., 2023 (5/75 cases) [20]	32	NA	11	Yes	Moderate-to-severe nuclear atypia	NR	Lung metastasis present at diagnosis
	47	NA	13	Extensive rhabdoid component	Infiltrative margins, serosal involvement	NR	Peritoneum (60 months)
	58	NA	7	Yes		NR	Peritoneum (144 months)
	68	NA	13	Yes	Infiltrative margins, serosal involvement	NR	Death (96 months) Peritoneum (60 months)
	73	NA	3	Yes	Infiltrative margins, 9/10 HPF, necrosis	NR	Death (50 months) Brain (30 months) Femur (48 months)
Bi et al., 2023 (7/22 cases, 1 already reported by Chen et al., 2021) [41]	33	Intramyometrial mass	2	Yes	Infiltrative margins	*GREB1–NCOA2* fusion	Death (177 months) Retroperitoneal recurrence with abdominal aorta involvement (167 months) Pelvic lymph nodes involvement presents at diagnosis
	48	Intramyometrial mass	13	Yes	Absent	*GREB1–NCOA2* fusion	Pelvis and omentum (45 months)
	38	Intramyometrial mass	NA	Yes	Infiltrative margins	*GREB1–NCOA2* fusion	Pelvis and omentum (101 months)
	48	Intramyometrial mass	3.5	Yes	Infiltrative margins	*GREB1–NCOA1* fusion	Pelvis (13 months). Peritoneal involvement present at diagnosis.
	65	Intramyometrial mass	15	Yes	Infiltrative margins	*GREB1–NCOA1* fusion	Death (44 months) Pelvis (35 months)
	40	Polypoid mass	4	Yes	Absent	*ESR1–NCOA2* fusion	Local recurrence (21 and 64 months)
	45	Intramyometrial mass, with cystic areas	8	Yes	Absent	*ESR1–NCOA3* fusion	Pelvis (56 months)
Xiong et al., 2023 (6/18 cases) [58]	41	NR	5.5	Yes	Infiltrative margins	*ESR1–NCOA2* fusion	Peritoneum (144.4 months)
	46	NR	2.5	Yes	Absent	Negative for *NCOA1–3* fusion and JAZF1/SUZ12/PHF1 rearrangement	Death (26.3 months) Pelvis, colon (2.5 months)
	19	NR	3	Yes	Absent	NA	Metastasis (site NA) (69.9 months)
	36	NR	1.5	Yes	Infiltrative margins, necrosis, significant nuclear atypia, 10/10 HPF	Negative for *NCOA1–3* fusion and JAZF1/SUZ12/PHF1 rearrangement	Pelvis, lung (56.5 months)
	55	NR	13	Yes	Infiltrative margins, significant nuclear atypia	*GREB1–NCOA2* fusion	Pelvis, colon (195.3 months)
	48	NR	2.2	Yes	Infiltrative margins	NA	Lung (21.1 months)

Table Legend: HPF: high-power field; LVI: lymphovascular invasion; NA: not available; NR: not reported.

## Data Availability

Not applicable.
